# Hemin attenuates response of primary rat adipocytes to adrenergic stimulation

**DOI:** 10.7717/peerj.12092

**Published:** 2021-09-02

**Authors:** Tomasz Szkudelski, Karina Frąckowiak, Katarzyna Szkudelska

**Affiliations:** 1 Department of Animal Physiology, Biochemistry and Biostructure, Poznań University of Life Sciences, Poznań, Poland

**Keywords:** Hemin, Adipocytes, Metabolism

## Abstract

Hemin is an activator of heme oxygenase-1 (HO-1), an enzyme catalyzing heme degradation. Up-regulation of HO-1 is observed in response to various pathological conditions. Moreover, pharmacological activation of HO-1 is associated with numerous beneficial effects in the organism. Hemin was shown to exert, among other, anti-diabetic and anti-obesity properties. These effects are strongly linked with adipose tissue. However, the direct influence of hemin on metabolism of the fat cells have not been explored. The present study aimed to determine the short-term effects of hemin on metabolism of the primary rat adipocytes. We focused on processes directly related to lipid accumulation, such as lipogenesis and lipolysis. For this purpose, the isolated cells were subjected for 2 h to 40 µM hemin, and effects of this compound on insulin-stimulated glucose conversion to lipids, lactate release, lipolysis induced by various stimuli, and also on the antilipolytic action of insulin were determined. It was shown that hemin did not affect insulin-induced lipogenesis and lactate release. However, hemin significantly decreased lipolysis stimulated by epinephrine. The inhibitory effect of hemin on epinephrine-induced lipolysis was not abolished in the presence of SnMP, an inhibitor of HO-1, which suggests hemin action irrespective of this enzyme. Similar inhibitory effects on epinephrine-induced lipolysis were observed in the presence of 3 and 12 mM glucose. Moreover, hemin was shown to reduce epinephrine-induced lipolysis also when glucose was replaced by alanine or by succinate. Apart from changes in epinephrine action, it was found that the lipolytic response of the adipocytes to isoproterenol was also diminished by hemin. However, hemin failed to affect lipolysis stimulated by dibutyryl-cAMP (a direct activator of protein kinase A), forskolin (an activator of adenylate cyclase), and also by DPCPX (an adenosine A_1_ receptor antagonist). Additionally, epinephrine-induced lipolysis was shown to be decreased by insulin, and this effect was deepened in the presence of hemin. These results indicate that short-term exposure of the adipocytes to hemin does not affect processes related to glucose metabolism, such as lipogenesis and lactate release. However, hemin was found to decrease the lipolytic response to adrenergic stimulation, which is associated with reduced lipid release from adipocytes. Moreover, our results indicate that hemin is also capable of diminishing the exaggerated lipolysis, which occurs in the presence of supraphysiological concentrations of glucose.

## Introduction

Hemin is a breakdown product of hemoglobin and is the oxidized form of heme. Hemin is one of the pharmacological activators of heme oxygenase-1 (HO-1), an enzyme which is involved in heme degradation. Mammalian heme oxygenase has two isoforms, HO-1 and HO-2. HO-1 is the inducible, and HO-2 the constitutive isoform. HO-1, known also as a heat shock protein 32, is up-regulated under various pathological conditions, such as oxidative and inflammatory stress, hypoxia, cytokine action, and also exposure to some xenobiotics or heavy metals. HO-1 is also activated in response to increased amounts of heme (Abraham & Kappas, 2008; Ndisang, 2010; Wegiel et al., 2014). Heme degradation, catalyzed by HO-1, is thought to be critical for cellular defense. This is associated with increased formation of heme-derived products, such as iron, CO and biliverdin. (Abraham & Kappas, 2008; Ndisang, 2010; Wegiel et al., 2014).

There is a large body of evidence that pharmacological induction of HO-1 has positive implications under various pathological conditions. Activation of HO-1 is well established to be associated with cardio-vascular benefits, exerts hepatoprotective and nephroprotective effects, and also reduces pulmonary disease and cancer (Abraham & Kappas, 2008; Wegiel et al., 2014). Moreover, pharmacological activation of HO-1 is known to reduce the inflammatory processes in various tissues (Abraham & Kappas, 2008; Ndisang, 2010). Rodent studies have shown that hemin treatment is also associated with anti-diabetic and anti-obesity effects (Ndisang, 2010; Ndisang & Jadhav, 2013; Ndisang, Jadhav & Mishra, 2014; Ju et al., 2014). Hemin administration to diabetic animals causes activation of HO-1 in metabolically active tissues, such as liver, the skeletal muscle, and also adipose tissue. Adipose tissue is well established to play a relevant role in the pathogenesis and progression of type 2 diabetes. White adipocytes store lipids, and also secret adipokines, which have multiple regulatory functions. The balance between lipogenesis and lipolysis is essential for avoiding the adipose tissue excess or deficit in the body. This is a significant issue given that increased adipocyte lipid accumulation leads to overweight or obesity and to insulin resistance and type 2 diabetes (Frühbeck et al., 2014; Luo & Liu, 2016).

There is a large body of evidence showing that hemin therapy is associated with reduced adiposity (Ndisang, 2010; Ndisang & Jadhav, 2013; Ndisang, Jadhav & Mishra, 2014; Ju et al., 2014). It was demonstrated that hemin administered to Zucker diabetic fatty (ZDF) rats decreases retroperitoneal adipocyte hypertrophy and adiposity, and also reduces inflammatory markers in perirenal adipose tissue (Ndisang, Jadhav & Mishra, 2014). Moreover, in mice fed a high-fat diet, hemin was demonstrated to reduce adipose tissue inflammation (Tu et al., 2014) and adiposity (Ndisang, Jadhav & Mishra, 2014). These data clearly show that hemin therapy is associated with many beneficial effects in adipose tissue. Moreover, results of *in vitro* studies indicate that hemin regulates preadipocyte maturation (Swierczewski et al., 1987; Chen & London, 1981; Moreno-Navarrete et al., 2017). Results of *in vivo* studies indicate that hemin treatment increases the expression and activity of HO-1. This is associated with changes covering other relevant intracellular molecules, such as glucose transporter-4 (GLUT4), AMP-activated protein kinase (AMPK), cAMP and cGMP in tissues of rats. However, all these alterations were shown to be abolished by inhibition of HO-1, which indicates that in this case hemin action is dependent of HO-1 (Ndisang, Lane & Jadhav, 2009). On the other hand, hemin may interact with many other compounds irrespectively of HO-1. Hemin has lipophilic properties and may interact, among others, with some cellular receptors (such as the nuclear hormone receptor REV-ERB involved in the regulation of circadian rhythm), with various proteins (including blood albumin) and with proteasome (participating in degradation of damaged proteins). Hemin may also interact with components of the cell membrane (Schaer et al., 2013). The ability of hemin to interact with various receptors and regulatory molecules suggests its possible influence also on hormones and molecules involved in the adipocyte metabolism. However, the direct effects of hemin on adipocyte metabolism have not been explored. The present study aimed to determine whether hemin affects lipogenesis and lipolysis in freshly isolated rat adipocytes.

## Materials & Methods

### Reagents

Hemin, D-glucose, L-alanine, mono-methyl succinate, epinephrine, isoproterenol, dibutyryl-cAMP, forskolin, DPCPX (8-Cyclopentyl-1,3-dipropylxanthine), insulin, MTT (3-(4,5-dimethylthiazol-2-yl)-2,5-diphenyltetrazolium bromide), collagenase (from *Clostridium histolyticum*, type II), reagents used to prepare Krebs-Ringer buffer (KRB; containing 118 mM NaCl, 4.8 mM KCl, 1.3 mM, CaCl_2_, 1.2 mM KH_2_PO_4_, 1.2 mM MgSO_4_, 24.8 mM, NaHCO_3_, 10 mM HEPES and 3% bovine serum albumin, fraction V), and also reagents used to determine concentrations of glycerol (KOH, isopropanol, anhydrous ammonium acetate, sodium meta-periodate, 2,4-pentadione) and lactate (lactate dehydrogenase, NAD^+^, glycine buffer) were obtained from Sigma-Aldrich (St Louis, MO, USA). SnMP (Tin Mesoporphyrin IX chloride) was purchased from Cayman Chemical (Michigan, USA). Dole’s extraction mixture used to stop lipogenesis, contained isopropanol-heptane-1N H_2_SO_4_(40:10:1). D-[U-^14^C]-glucose (250 mCi/2.5 ml water) was obtained from Hartmann Analytics GmbH, and scintillation cocktail (OptiPhase HiSafe 3) from Perkin Elmer.

### Animals

Male Wistar rats were used in the study. Rats were obtained from Mossakowski Medical Research Centre Polish Academy of Sciences in Warsaw (Poland). Animals were maintained in cages in an air-conditioned animal room with a constant temperature of 21 °C and with 12:12-hour dark-light cycle. Animals had free access to drinking water and to the standard laboratory diet (Labofeed B, “Morawski”, Kcynia, Poland). According to Polish law, agreement of the Local Ethical Commission for Investigations on Animals was not required, because tissues were collected after the death and no experiments on alive animals were performed.

### Isolation of adipocytes

In the present study, we used freshly isolated epididymal adipocytes, which are metabolically very active. Moreover, these cells are suitable to study short-term hormonal regulation of metabolism (Rodbell, 1964). The adipocytes were isolated from rats weighing 300-320 g. Just before tissue sampling, animals were killed by decapitation using a laboratory guillotine. After decapitation of rats, the epididymal fat tissue was used for cell isolation. The adipocytes were isolated according to the method described by Rodbell (1964) with some modifications (Szkudelska, Okulicz & Szkudelski, 2021). The tissue was rinsed with saline, placed in a plastic flask and cut-down with scissors. For the adipocyte isolation, Krebs-Ringer buffer containing 3 mM glucose and 1 mg/ml collagenase was used. Before use, the buffer was gassed with a mixture of O_2_ and CO_2_ (95% and 5%, respectively), and its pH was adjusted to 7.4. The fat tissue was incubated in a plastic flask containing this buffer at 37 °C for 60 min and with a gentle shaking. After the end of incubation, the adipocytes were filtered using a nylon mesh and were precisely rinsed with the buffer without collagenase. Afterwards, the cells were transferred to the plastic tubes and were left for flotation. Then, the aliquots of the freshly isolated adipocytes with the buffer were taken for the appropriate experiments.

### Effects of hemin on lipogenesis and lactate release

In order to study glucose conversion to lipids, the adipocytes were incubated in the plastic tubes with the KRB containing 3 mM glucose, D-[U-^14^C]-glucose (0.5 µCi per tube) without insulin (basal lipogenesis) or in the presence of 10 nM insulin (stimulated lipogenesis). To study effects of hemin on basal lipogenesis, the adipocytes were incubated without insulin and in the presence of 40 µM hemin. In the case of insulin-induced lipogenesis, the adipocytes were incubated in the tubes with the KRB containing 3 mM glucose, D-[U-^14^C]-glucose, 10 nM insulin and hemin. All incubations were performed for 2 h at 37 °C and with a gentle shaking. After this time, 5 ml of cold Dole’s extraction mixture was added and mixed to stop lipogenesis (Dole & Meinertz, 1960). Then, the tubes were shaken, 2 ml of water and 3 ml heptane were added, and tubes were mixed once again. Lastly, the upper phase was transferred into the scintillation vials containing the scintillation cocktail and the radioactivity of total lipids was measured using a *β*-counter. In this method, the amounts of glucose conversion to total lipids reflects the intensity of lipogenesis (Rodbell, 1964).

To study lactate release, the adipocytes were incubated in the KRB containing 3 mM glucose and 10 nM insulin without hemin or in the presence of 40 µM hemin. The cells were incubated for 2 h at 37 °C with a gentle shaking. After this time, the adipocytes were removed, and aliquots of the buffer were mixed with 10% trichloroacetic acid to remove proteins. Then, tubes were centrifuged and supernatant was used for analysis. The enzymatic method with lactate dehydrogenase was applied in the study. In this method, lactate in the presence of NAD is converted by lactate dehydrogenase to pyruvate and NADH. Aliquots of deproteinized KRB were mixed in tubes containing glycine buffer, lactate dehydrogenase and NAD. Then, tubes were incubated at 37 °C for 15 min. After the incubations, samples were cooled, and the absorbance of NADH generated from NAD^+^ and reflecting concentration of lactate was read at 340 nm (Everse, 1975).

In the case of studies concerning lipogenesis and lactate release, 10^6^ adipocytes were incubated in 1 ml of KRB without hemin or in the presence of 40 µM hemin.

### Effects of hemin on lipolysis

In the present study, effects of hemin on basal lipolysis and lipolysis induced by different lipolytic stimuli were compared. In the case of basal lipolysis, the adipocytes were placed in the KRB and were exposed to 40 µM hemin without lipolytic agents. In the first set of our experiment, epinephrine was added to stimulate lipolysis and adipocytes were incubated in the KRB containing 0.5 µM epinephrine alone or epinephrine with 40 µM hemin. A part of the adipocytes used in the study was preincubated with 20 µM SnMP alone (an inhibitor of HO-1). After 30 min of preincubation, these cells were further incubated in the medium containing 0.5 µM epinephrine and SnMP or epinephrine, SnMP and hemin. Then, epinephrine was replaced by isoproterenol and cells were exposed to 0.5 nM isoproterenol alone or isoproterenol in the combination with 40 µM hemin. Effects of hemin on epinephrine-induced lipolysis were also explored in the absence of glucose. In this part of the experiment, the adipocytes were incubated in the buffer containing 6 mM alanine and 0.5 µM epinephrine with or without 40 µM hemin. Moreover, the fat cells were also subjected to 6 mM succinate and 0.5 µM epinephrine alone or in the presence of 40 µM hemin. In our study, the cells were also incubated with forskolin to stimulate the lipolytic process. For this purpose, the adipocytes were subjected to 1 µM forskolin alone or in the presence of 40 µM hemin. Moreover, dibutyryl-cAMP (DB-cAMP) was also used to induce lipolysis. In this case, the adipocytes were incubated in the buffer containing 0.25 mM DB-cAMP alone or with 40 µM hemin. Experiments in which DPCPX was used to induce lipolysis were also done. In this part of the study, the fat cells were exposed to 0.5 µM DPCPX without hemin or in the presence of 40 µM hemin. All these incubations (in addition to incubations with alanine and succinate) were performed in the KRB containing 3 mM glucose.

Additionally, effects of hemin on lipolysis in the presence of supraphysiological concentration of glucose were also studied. For this purpose, the adipocytes were placed in the buffer containing 12 mM glucose and 0.5 µM epinephrine with or without 40 µM hemin.

### Effects of hemin on anti-lipolysis

Apart from the lipolytic response, effects of hemin on the anti-lipolytic action of insulin in the primary rat adipocytes were also explored. The isolated cells were incubated with 0.5 µM epinephrine and epinephrine in the presence of 10 nM insulin with or without 40 µM hemin. In order to compare effects of hemin in the presence of physiological and supraphysiological concentrations of glucose, the isolated cells were exposed to epinephrine, insulin and hemin in the buffer containing 3 or 12 mM glucose.

In studies concerning lipolysis and anti-lipolysis, 10^6^ adipocytes were incubated for 2 h at 37 °C with a gentle shaking in 1 ml of KRB with 40 µM hemin. Moreover, in each case, incubations without lipolytic agents were also made to study basal lipolysis.

### Glycerol determination

At the end of incubations, glycerol release from the adipocytes to the buffer was measured. Glycerol release reflects the intensity of the lipolytic process. First, the adipocytes were removed and aliquots of the incubation buffer were transferred to Eppendorf tubes and mixed with 10% trichloroacetic acid to precipitate proteins. Then, tubes were vortexed and centrifuged, and supernatant was used for analysis. In the present study, the colorimetric Hantzsch condensation method was used according to the description of Foster & Dunn (1973) with some modifications (Szkudelska, Okulicz & Szkudelski, 2021). In this method, glycerol, in the presence of sodium meta-periodate, is oxidized to formaldehyde. Formaldehyde condenses with ammonia and acetylacetone to give a yellow product (3,5-diacetyl-1,4-dihydrolutidine) the absorbance of which is measured at 410 nm.

### Adipocyte viability

Effects of hemin on the adipocyte viability was determined using MTT test. For this purpose, the isolated cells (10^6^/ml) were incubated for 2 h in the KRB containing 3 mM glucose alone or glucose in the presence of 40 µM hemin. After this time, the cells preincubated with hemin were washed with the buffer containing glucose without hemin. Then, all adipocytes were incubated in the buffer containing 3 mM glucose with 0.5 mg/ml MTT. MTT in living cells is converted to formazan. After 1 h of incubation, 1 ml of isopropanol was added to each tube, tubes were shaken and centrifuged. Afterwards, the absorbance of formazan was read at 560 nm (Plumb, 2004). All incubations were performed at 37 °C with a gentle shaking.

### Statistical analysis

All results represent the mean ±  SEM from three independent experiments performed in 5 repetitions. The obtained results were evaluated statistically using analysis of variance and Tukey’s multiple range test. The differences were considered statistically significant at *p* < 0.05.

## Results

### Effects of hemin on lipogenesis and lactate release

**Figure 1:**
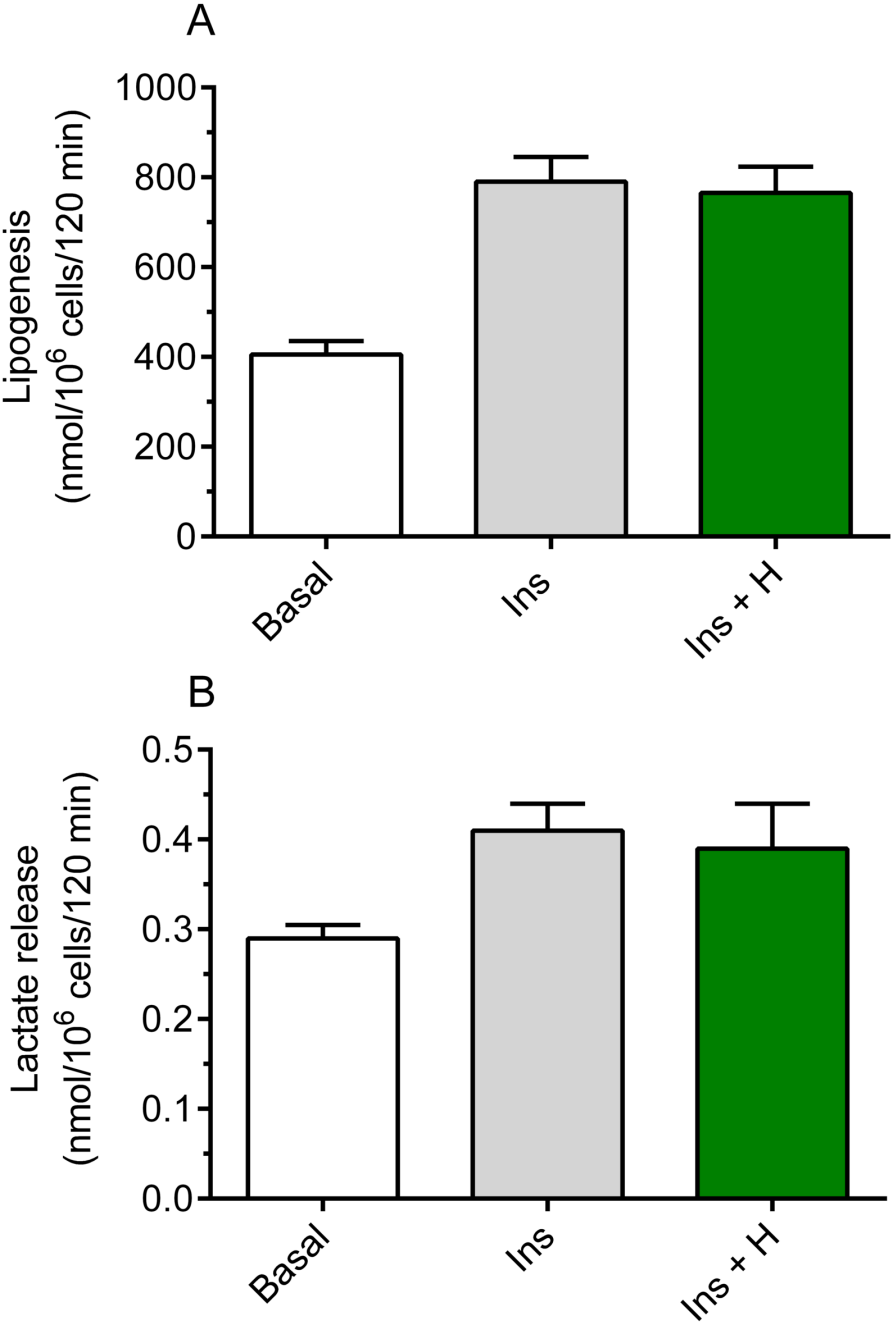
Effects of hemin on lipogenesis (A) and lactate release (B) in the isolated rat adipocytes. (A) The adipocytes were incubated in the presence of 3 mM glucose, D-[U-^14^C]-glucose without insulin (basal lipogenesis) or with 10 nM insulin (Ins; stimulated lipogenesis) and with 40 µM hemin (H). After 2 h of incubation, the cells were washed, total lipids were extracted and their radioactivity was measured. (B) The adipocytes were incubated in the presence of 3 mM glucose without insulin (basal release) or with 10 nM insulin (Ins; stimulated release) and with 40 µM hemin (H). After 2 h of incubation, the cells were removed and concentration of lactate released from adipocytes to the incubation medium was determined using the method with lactate dehydrogenase. Results represent the means ± SEM of 15 values taken from three independent experiments. The means are statistically different (*p* < 0.05) between basal and all remaining values.

It was shown that hemin failed to significantly affect basal lipogenesis (the mean basal glucose conversion to lipids was 420 ±  29 nmol/10^6^ cells/120 min without hemin and 442 ±  33 nmol/10^6^ cells/120 min in the presence of hemin). It was also demonstrated that insulin substantially increased glucose conversion to lipids in the freshly isolated rat adipocytes, compared with basal (non-stimulated) lipogenesis. Insulin was shown to enhance lipogenesis by 95%, and the rise was statistically significant (*p* < 0.05; Fig. 1A). However, our study have shown that the adipocyte exposure to 40 µM hemin did not significantly affect insulin-induced glucose conversion to lipids (Fig. 1A).

Insulin was shown to significantly (*p* < 0.05) increase (by 41%) lactate release from the fat cells to the incubation medium, compared with basal release (Fig. 1B). It was also revealed that, similarly to effects related to lipogenesis, lactate release was not significantly changed in the presence of hemin (Fig. 1B).

### Effects of hemin on lipolysis

**Figure 2:**
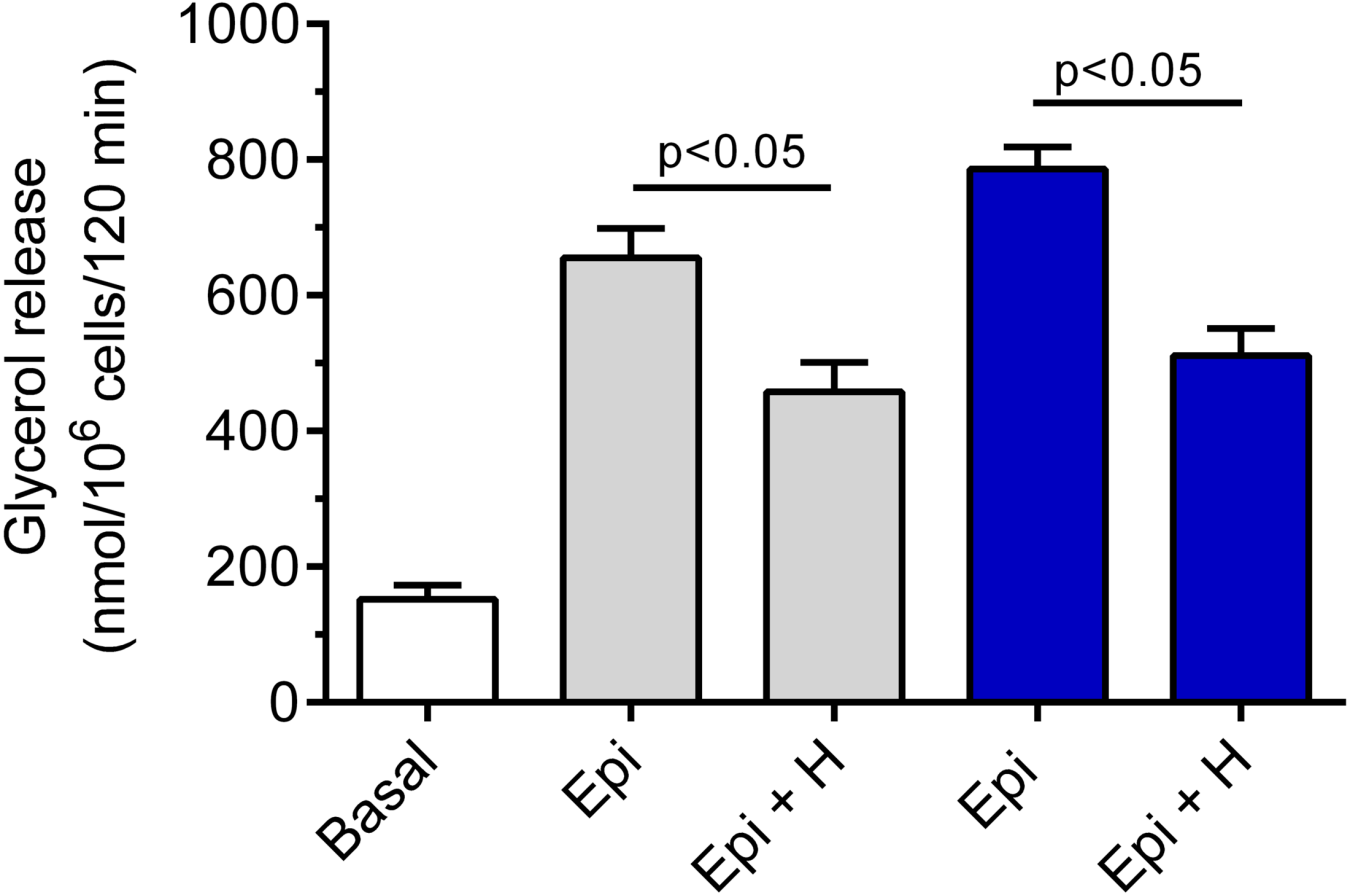
Effects of hemin on epinephrine-induced lipolysis in the isolated rat adipocytes in the presence of 3 or 12 mM glucose. The adipocytes were incubated without epinephrine (basal lipolysis), with 0.5 µM epinephrine (Epi), 3 mM glucose (grey bars) or 0.5 µM epinephrine, 12 mM glucose (blue bars) and with or without 40 µM hemin (H). After 2 h of incubation, the cells were removed and concentrations of glycerol released from the adipocytes to the incubation medium were determined. Results represent the means ±  SEM of 15 values taken from three independent experiments. The means are also statistically different (*p* < 0.05) between basal and all remaining values.

Hemin was demonstrated to be ineffective in relation to basal lipolysis (the mean basal glycerol release was 178 ± 12 nmol/10^6^ cells/120 min without hemin and 166 ± 19 nmol/10^6^ cells/120 min in the presence of hemin). The adipocyte exposure to epinephrine was associated with a significant (*p* < 0.05) rise in glycerol release, compared with basal values. Epinephrine increased lipolysis by 330% and by 417% in the presence of 3 and 12 mM glucose, respectively (Fig. 2). Our study have shown that the lipolytic response of the adipocytes to epinephrine stimulation was markedly affected by hemin. Hemin was demonstrated to evoke a clear-cut decrease in epinephrine-stimulated lipolysis. This decrease was statistically significant (*p* < 0.05) and was 30% in the presence of 3 mM glucose, compared with lipolysis observed without hemin (Fig. 2). Moreover, it was shown that the adipocyte exposure to epinephrine and hemin in the presence of 12 mM glucose also caused a significant (*p* < 0.05) reduction in glycerol release. In this case, lipolysis was lower by 35% (Fig. 2).

**Figure 3:**
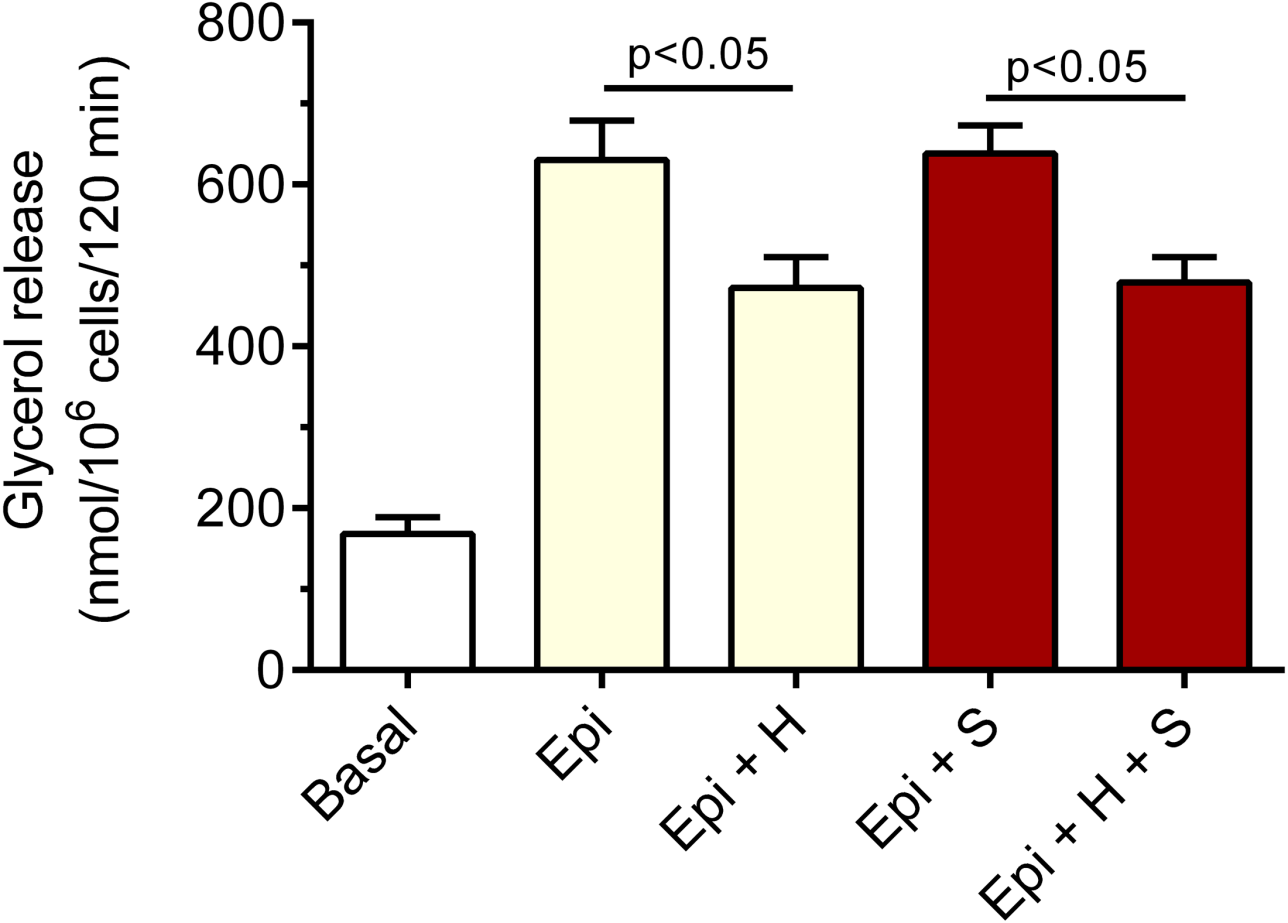
Effects of hemin and SnMP on epinephrine-induced lipolysis in the isolated rat adipocytes in the presence of 3 mM glucose. A part of the adipocytes were preincubated for 30 min with 20 µM SnMP, and then cells were incubated in the presence of 3 mM glucose without epinephrine (basal lipolysis), with 0.5 µM epinephrine (Epi), epinephrine with 40 µM hemin (Epi+H), epinephrine with 20 µM SnM (Epi + S) or epinephrine, hemin and SnMP (Epi + H + S). After 2 h of incubations, the cells were removed and concentrations of glycerol released from the adipocytes to the incubation medium were determined. Results represent the means ± SEM of 15 values taken from three independent experiments. The means are also statistically different (*p* < 0.05) between basal and all remaining values.

We have also revealed that the inhibitory effect of 40 µM hemin on epinephrine-induced lipolysis studied in the presence of 3 mM glucose was not significantly affected by 20 µM SnMP. Hemin reduced epinephrine-stimulated lipolysis by 25% and by 27% in the presence of SnMP (Fig. 3). In both cases effects were statistically significant (*p* < 0.05), compared with epinephrine alone. However, the difference between effects of hemin and hemin with SnMP was small and was not statistically significant. Effects elicited by SnMP on epinephrine-induced glycerol release were also negligible (Fig. 3).

**Figure 4:**
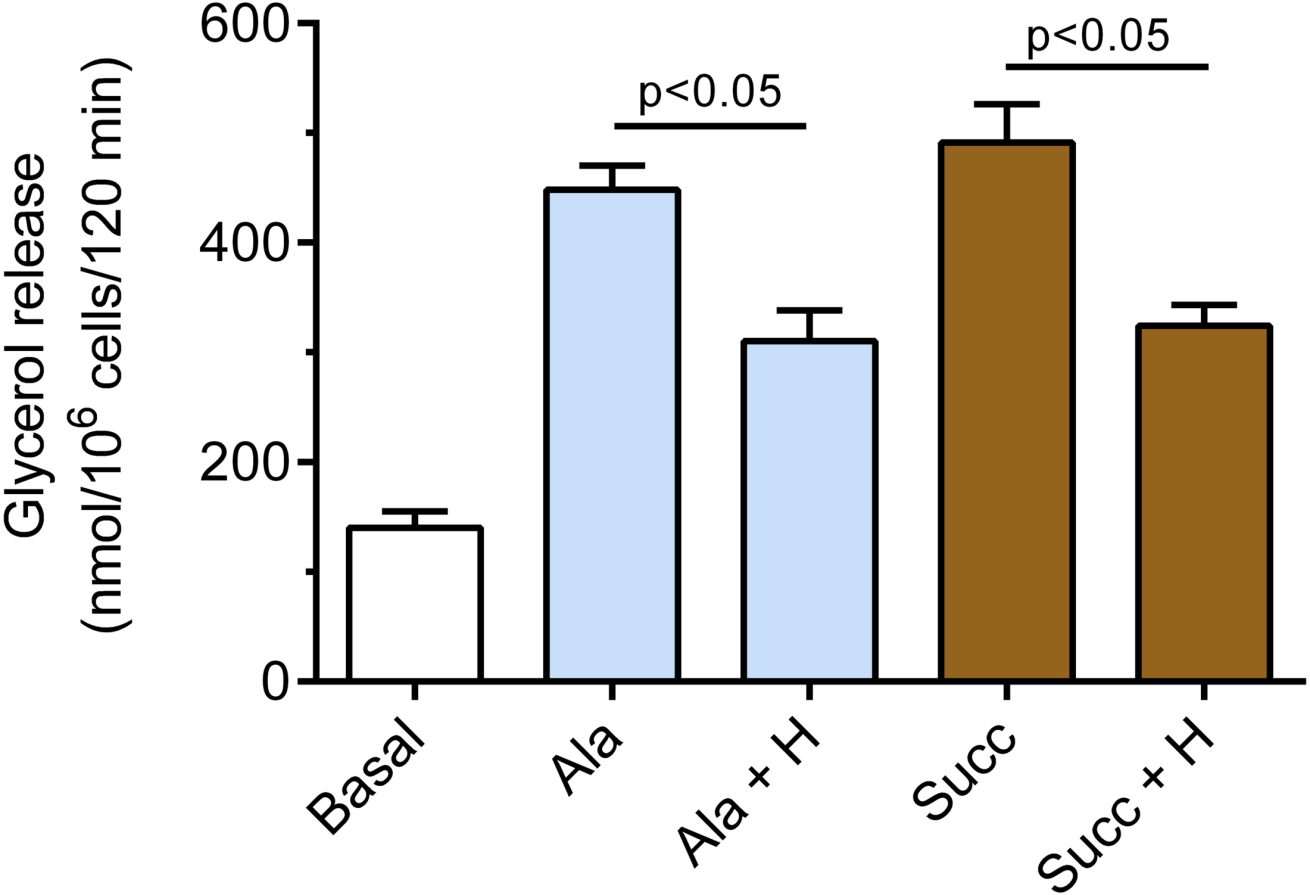
Effects of hemin on epinephrine-induced lipolysis in the isolated rat adipocytes in the presence of alanine or succinate. The adipocytes were incubated without epinephrine (basal lipolysis), with 0.5 µM epinephrine, 6 mM alanine (Ala) or 0.5 µM epinephrine, 6 mM succinate (Succ) and with or without 40 µM hemin (H). After 2 h of incubation, the cells were removed and concentrations of glycerol released from the adipocytes to the incubation medium were determined. Results represent the means ± SEM of 15 values taken from three independent experiments. The means are also statistically different (*p* < 0.05) between basal and all remaining values.

It was also demonstrated that hemin affects epinephrine-induced lipolysis in the presence of alanine. Exposure of the adipocytes to hemin evoked a marked decrease in the lipolytic process. The inhibitory effect of hemin was statistically significant (*p* < 0.05), and lipolysis was diminished by 31% (Fig. 4). Moreover, it was found that hemin significantly (*p* < 0.05) reduced the lipolytic response to epinephrine in the presence of succinate. In the buffer containing succinate, hemin decreased lipolysis by 34% (Fig. 4).

**Figure 5:**
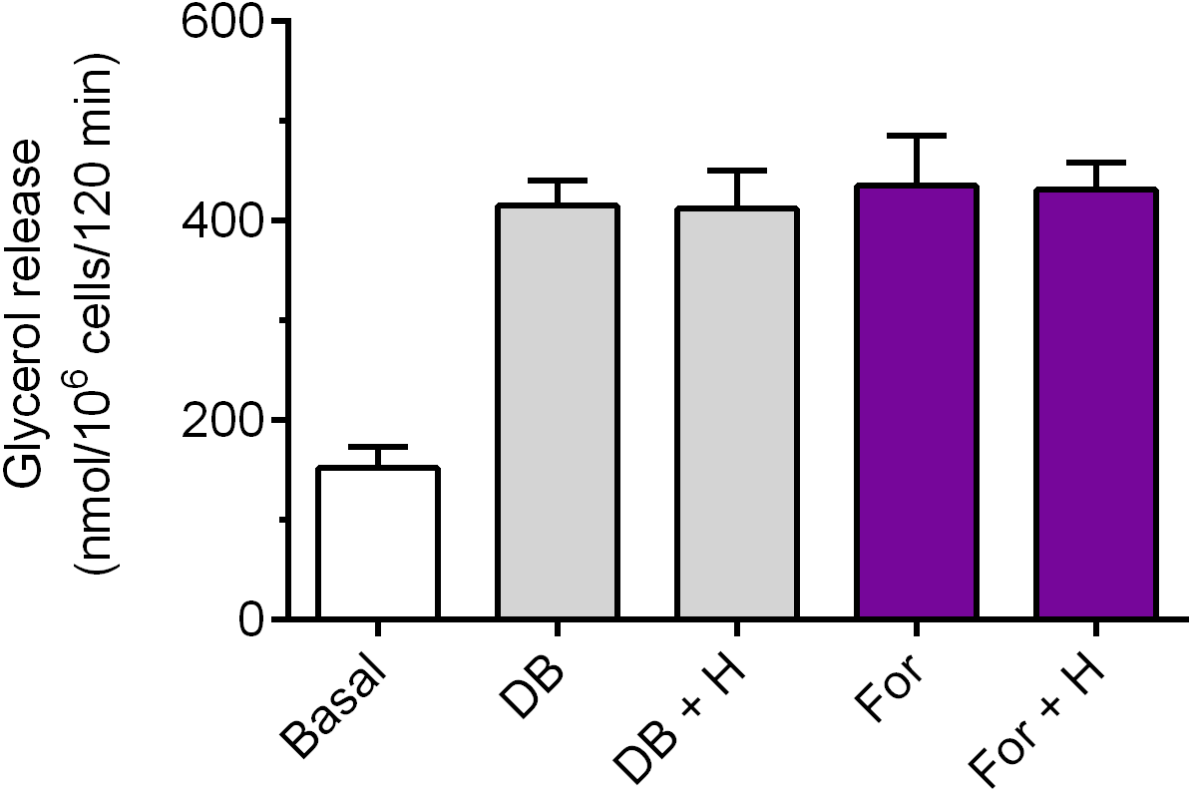
Effects of hemin on lipolysis induced by dibutyryl-cAMP or forskolin in the isolated rat adipocytes. The adipocytes were incubated without lipolytic stimuli (basal lipolysis), with 0.25 mM dibutyryl-cAMP (DB) or 1 µM forskolin (For) and with or without 40 µM hemin (H). After 2 h of incubation, the cells were removed and concentrations of glycerol released from the adipocytes to the incubation medium were determined. Results represent the means ± SEM of 15 values taken from three independent experiments. The means are statistically different (*p* < 0.05) between basal and all remaining values.

Exposure of the adipocytes to DB-cAMP caused a marked increase in glycerol release, compared with control cells. This rise was by 173% and was statistically significant (*p* < 0.05; Fig. 5). It was demonstrated that lipolysis triggered by DB-cAMP was not significantly affected in the presence of hemin (Fig. 5). The adipocyte exposure to forskolin also enhanced glycerol release. The potentiatory effect of forskolin was significant (*p* < 0.05) and was 186% (Fig. 5). However, incubations of the adipocytes with forskolin in the combination with hemin did not induce any significant changes in the lipolytic process, compared with forskolin alone (Fig. 5).

**Figure 6:**
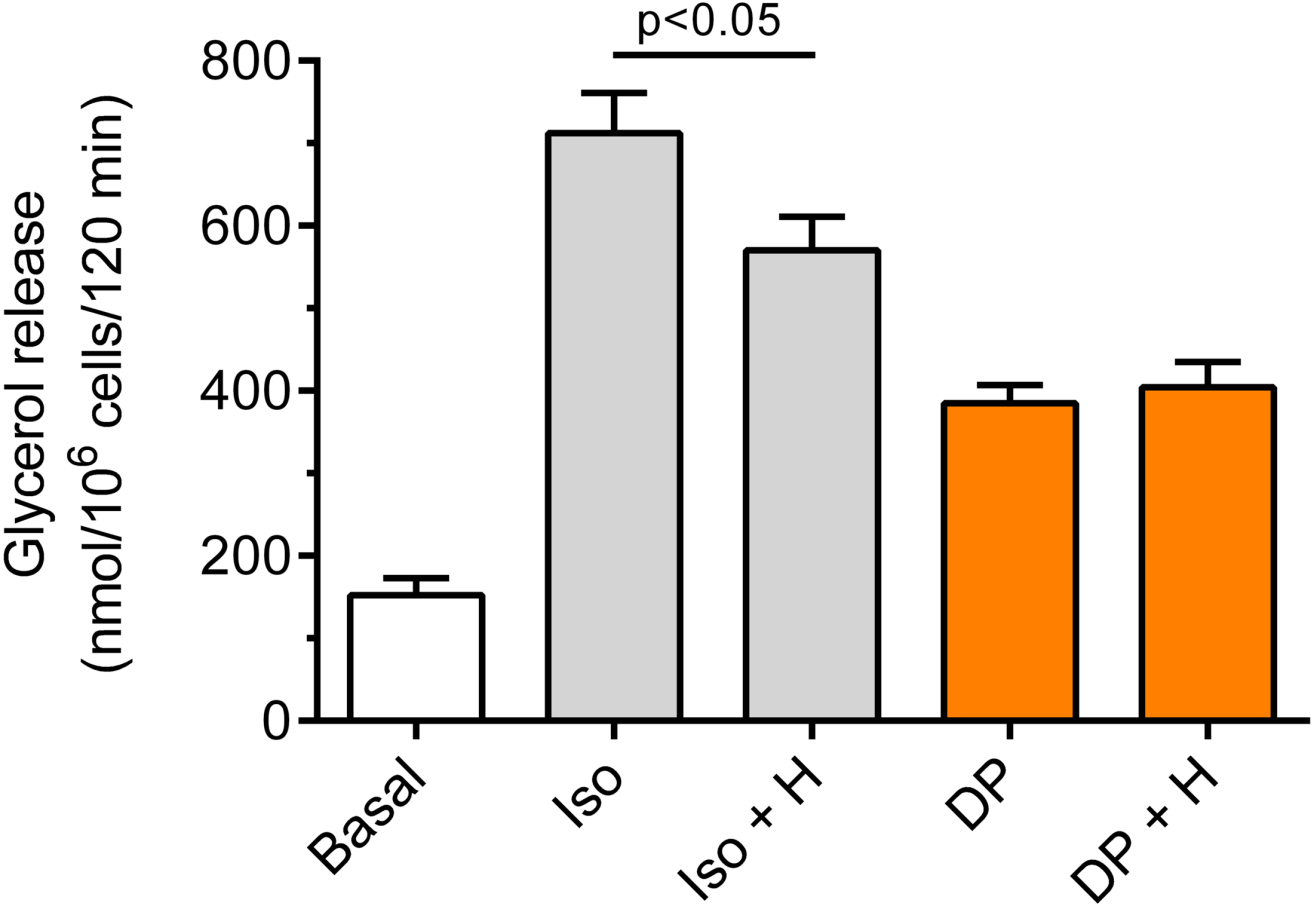
Effects of hemin on lipolysis induced by isoproterenol or DPCPX in the isolated rat adipocytes. The adipocytes were incubated without lipolytic stimuli (basal lipolysis), with 0.5 nM isoproterenol (Iso) or 0.5 µM DPCPX (DP) and with or without 40 µM hemin (H). After 2 h of incubation, the cells were removed and concentrations of glycerol released from the adipocytes to the incubation medium were determined. Results represent the means ± SEM of 15 values taken from three independent experiments. The means are also statistically different (*p* < 0.05) between basal and all remaining values.

Our study have shown that in the freshly isolated rat adipocytes subjected to isoproterenol, a significant (*p* < 0.05) rise in lipolysis was found, compared with non-stimulated conditions. The increase in glycerol release induced by isoproterenol was 368% (Fig. 6). Moreover, it was found that the lipolytic response of the adipocytes to isoproterenol was significantly (*p* < 0.05; Fig. 6) attenuated in the presence of hemin. In this case, hemin decreased glycerol release by 24% (Fig. 6).

It was also shown that the adipocyte exposure to DPCPX substantially increased lipolysis. In the presence of DPCPX, lipolysis was higher by 153%, and this effect was statistically significant (*p* < 0.05). However, lipolysis induced by DPCPX was not significantly altered by hemin (Fig. 6).

### Effects of hemin on anti-lipolysis

**Figure 7:**
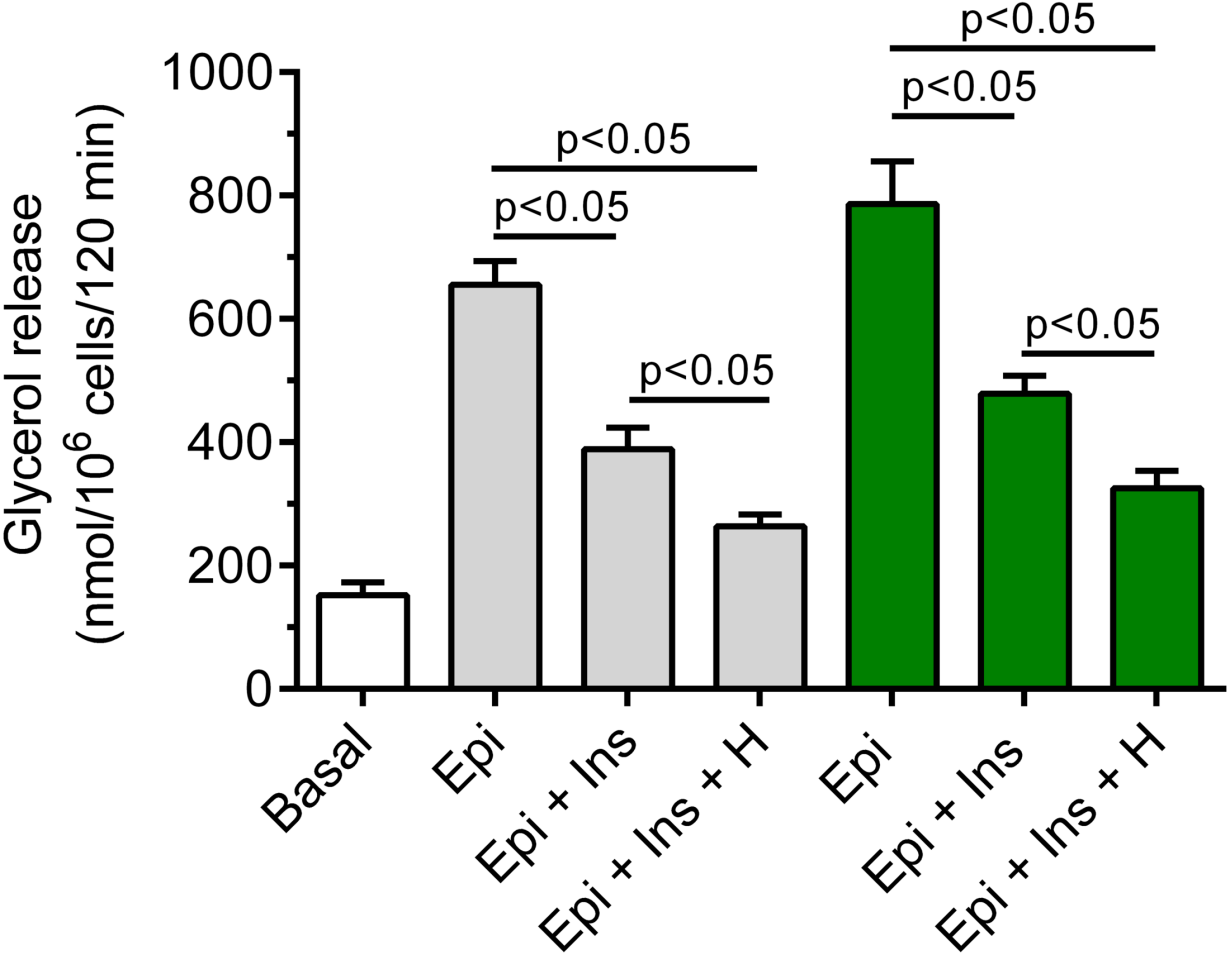
Effects of hemin on anti-lipolytic action of insulin in the presence of 3 or 12 mM glucose in the isolated rat adipocytes. The adipocytes were incubated without epinephrine (basal lipolysis), with 0.5 µM epinephrine (Epi), 0.5 µM epinephrine with 10 nM insulin (Ins + Epi) in the presence of 3 mM glucose (grey bars) or 12 mM glucose (green bars) with or without 40 µM hemin (H). After 2 h of incubation, the cells were removed and concentrations of glycerol released from the adipocytes to the incubation medium were determined. Results represent the means ± SEM of 15 values taken from three independent experiments. The means are also statistically different (*p* < 0.05) between basal and all remaining values.

It was shown that insulin markedly blunted the lipolytic response of the adipocytes to epinephrine. In the presence of 3 mM glucose, lipolysis was significantly (*p* < 0.05) lower in the adipocytes exposed to epinephrine and insulin, compared with effects of epinephrine alone. The inhibitory effect was 40% (Fig. 7). It was also found that exposure of the isolated rat adipocytes to hemin was associated with a further decrease in glycerol release. The inhibition caused by hemin was statistically significant (*p* < 0.05) and was 30% (Fig. 7).

It was also demonstrated that insulin evoked a clear-cut decrease in epinephrine-stimulated lipolysis in the cells incubated in the buffer containing 12 mM glucose. This effect was statistically significant (*p* < 0.05) and was 39%, compared with incubations without insulin (Fig. 7). Moreover, we have shown that glycerol release from the adipocytes incubated in the presence 12 mM glucose, epinephrine and insulin was markedly reduced by hemin. It was found that hemin decreased lipolysis by 32%, and its action was statistically significant (*p* < 0.05) (Fig. 7).

### Adipocyte viability

It was shown that formazan formation from MTT in the cells subjected for 2 h to 40 µM hemin or incubated without this compound did not differ significantly. The mean absorbance of formazan was 0.227 ± 0.09 and 0.219 ± 0.12 in the case of the control adipocytes and the cells treated with hemin, respectively.

## Discussion

Adipocyte lipid accumulation covers two pivotal processes, *i.e.,* lipogenesis and lipolysis. The present study have shown that lipid synthesis from glucose in the rat adipocytes was not significantly affected by 2-hour exposure to 40 µM hemin. Formation of lipids is the major pathway of glucose metabolism in the fat cells. This process was studied in the presence of insulin, which is the main physiological stimulator of lipogenesis. Insulin-induced lipogenesis is preceded by intracellular glucose transport *via* glucose transporter GLUT4, followed by sugar metabolism and formation of triglycerides. Along with lipogenesis, effects of hemin on lactate release were also explored. Intracellular lactate formation is strongly linked with a non-oxidative glucose metabolism. Our results have shown that hemin did not affect lactate release from the adipose cells. Other studies have revealed that hemin reduces adipogenesis, and also decreases intracellular glucose transport and expression of GLUT4 in adipocytes. These effects were, however, observed, after prolonged incubations with hemin (Moreno-Navarrete et al., 2017). On the other hand, exposure of human adipocytes to hemin for 24–48 h failed to affect adiponectin expression and secretion (Yang et al., 2015). Results of the present study indicate that pathways of glucose metabolism related to lipogenesis and lactate formation are unchanged after short-term exposure of the primary rat adipocytes to hemin.

On the other hand, we have shown a substantial influence of hemin on adipocyte lipolysis. Lipolysis was studied in the presence of epinephrine, one of the physiological stimulators of this process. Epinephrine-induced lipolysis was found to be substantially reduced in the fat cells subjected to hemin. Given that various factors have been implicated in the regulation of lipid decomposition (Frühbeck et al., 2014), hemin action may result from different causes. Adipocyte lipolysis is known to be an energy-dependent process and is decreased, when glucose supply and/or metabolism is reduced (Naito & Okada, 1975; Szkudelski & Szkudelska, 2000; Szkudelski & Szkudelska, 2017). In order to explore, whether the inhibition of epinephrine-induced lipolysis by hemin may be associated with reduced glucose transport and/or metabolism, glucose was replaced by alanine or by succinate. These compounds partially differ in relation to their intracellular transport and metabolism. In the absence of insulin, glucose is transported into adipocytes *via* GLUT1, alanine reaches the fat cells by the amino acid transport system, undergoes deamination and is metabolized in the mitochondria (Hatanaka et al., 2006), whereas mono-methyl succinate easily penetrates the adipocytes and is also metabolized in the mitochondria. Epinephrine-stimulated lipolysis studied in the presence of alanine or succinate was shown to be markedly reduced by hemin. These results demonstrate that hemin is capable of decreasing epinephrine-induced lipolysis also in the absence of glucose. Moreover, this indicates that hemin action is not associated with inhibition of glucose transport or its metabolism. This is in line with our findings that hemin did not affect glucose conversion to lipids.

Adipocyte lipolysis is known to be increased in the presence of higher concentrations of glucose (Naito & Okada, 1975; Szkudelski & Szkudelska, 2000; Szkudelski & Szkudelska, 2017). This is associated with enhanced release of glycerol and non-esterified fatty acids from the fat cells. It was also shown that an exaggerated lipolysis stimulated by epinephrine under conditions of short-term exposure to supraphysiological concentrations of glucose is inhibited by insulin (Szkudelski & Szkudelska, 2000), the major physiological anti-lipolytic hormone (Frühbeck et al., 2014). Therefore, effects of hemin on the lipolytic response to epinephrine were studied in the adipocytes subjected to a high concentration of glucose. It was found that hemin markedly reduced epinephrine-induced lipid breakdown in the fat cells incubated with 12 mM glucose. This indicates that the excessive lipolysis, which occurs in the presence of supraphysiological concentration of glucose is effectively alleviated by hemin.

Long-term action of hemin is known to be associated with up-regulation of HO-1 in the adipose cells. Hemin was found to increase the expression of HO-1 in human (Yang et al., 2015; Moreno-Navarrete et al., 2017) and mice (Tu et al., 2014) adipocytes. However, these effects were shown after more prolonged (24-48 h) exposure to hemin. Results of the present study suggest that short-term influence of hemin on epinephrine-induced lipolysis is not due to activation of HO-1. This assumption is strongly supported by our findings showing that effect of hemin on epinephrine action were not abolished by SnMP, an inhibitor of HO-1. Moreover, other studies indicate that HO-1 is not a sole hemin target (Schaer et al., 2013).

Epinephrine promotes lipolysis *via* the sequence of events involving its binding to adrenergic receptor, activation of G_s_ protein, induction of adenylate cyclase, a rise in cAMP content, activation of PKA, and also phosphorylation of perilipins and intracellular lipases (Frühbeck et al., 2014). Hemin can reduce epinephrine-induced lipolysis affecting different steps of the lipolytic cascade. In order to better precise hemin action, epinephrine was replaced by dibutyryl-cAMP, a direct activator of PKA. It was demonstrated that the lipolytic response of the adipocytes to dibutyryl-cAMP was unchanged in the presence of hemin. This indicates that the inhibitory effects of hemin on epinephrine-induced lipolysis cover steps before PKA. Moreover, the adipocytes were subjected to forskolin, which directly activates adenylate cyclase and thereby triggers lipolysis. Our results have shown that forskolin-induced lipolysis was not significantly affected by hemin. This strongly suggests that the action of hemin on lipolysis evoked by epinephrine involves interaction with adrenergic receptor, its ligand or Gs protein. Adrenergic receptor has many subtypes. Epinephrine is a non-selective agonist and may interact with five subtypes, *i.e., α*_1_, *α*_2_, *β*_1_, *β*_2_ and *β*_3_. Therefore, isoproterenol was used, a pharmacological agonist, which is more selective and activates only *β*_1_ and *β*_2_ adrenergic receptors. It was shown that the lipolytic process induced by isoproterenol was substantially diminished by hemin. This is in line with results concerning epinephrine action and indicates that hemin acts *via* adrenergic receptor or Gs protein.

**Scheme 1:**
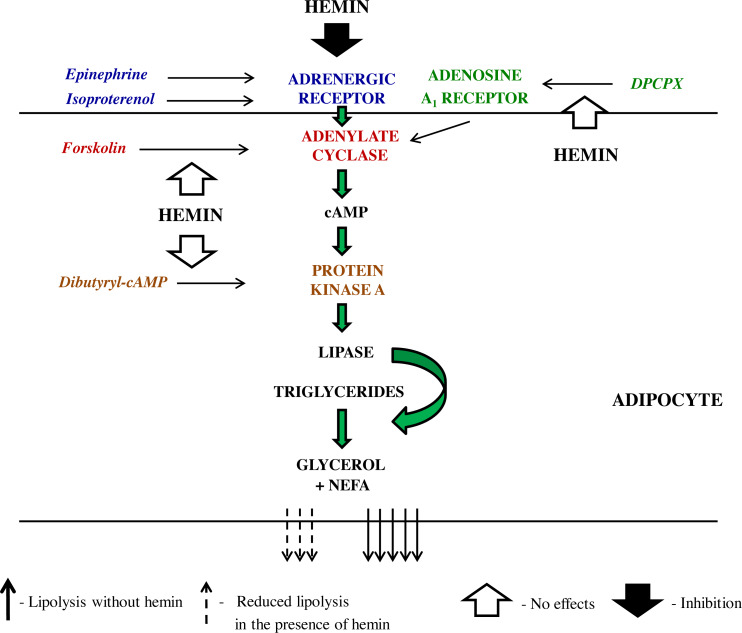
The schematic representation of the action of lipolytic agents used in the study and effects induced by hemin in the rat adipocytes.

Along with the adrenergic regulation, the lipolytic activity of the adipocytes is also largely influenced by adenosine. Adenosine is tonically released from these cells and binds to adenosine A_1_ receptor, which is followed by the inhibition of lipolysis. On the other hand, a blockade of adenosine A_1_ receptor markedly enhances this process (Szkudelski, Szkudelska & Nogowski, 2009; Frühbeck et al., 2014). This is a relevant regulation given that an abnormal adenosine A_1_ receptor signaling contributes to some metabolic diseases (Dhalla et al., 2009). Therefore, effects of hemin on lipolysis induced by DPCPX, an adenosine A_1_ receptor antagonist, were also explored. It was shown that adipocyte exposure to DPCPX substantially increased the lipolytic process. However, lipolysis triggered by DPCPX was unaltered in the presence of hemin. This indicates that the inhibitory effect of adenosine is not affected by hemin. Effects of hemin on lipolysis, that have been revealed in the present study are shown on Scheme 1.

Apart from lipolysis, effects of hemin on the anti-lipolytic action of insulin in the isolated rat adipocytes were also explored. Since insulin does not affect basal lipolysis, the isolated adipocytes were exposed to epinephrine in order to stimulate lipolysis. As expected, insulin significantly reduced the lipolytic response of the adipocytes to epinephrine. The insulin action is largely associated with activation of phosphodiesterase 3B, the main enzyme catalyzing cAMP degradation in the fat cells. This is followed by a reduction in cAMP levels and a reduction in lipolysis (Degerman et al., 2011; Frühbeck et al., 2014). It was shown that in the adipocytes subjected to epinephrine and insulin, lipolysis was further decreased by hemin. We have shown that hemin reduced epinephrine-induced lipolysis in the isolated rat adipocytes. Given that hemin in the present study reduced the lipolytic response of the adipocytes to epinephrine itself, the observed decrease may be supposed to result from the effects of hemin on epinephrine action, and not from alterations in insulin action. This indicates that hemin does not affect the anti-lipolytic action of insulin. These results are in accord with our findings showing that hemin is ineffective in relation to insulin-induced lipogenesis. Effects of hemin on the antilipolytic action of insulin were compared in the presence of physiological and supraphysiological concentrations of glucose. It was shown that hemin action is similar in adipocytes subjected to low and high concentrations of glucose.

The effective concentrations of hemin and the term of exposure, that are applied *in vitro* are very differentiated. In the present study, the adipocytes were subjected for 2 h to 40 µM hemin. This is relatively a short-term exposure, compared with experiments on gene expression or adipocyte maturation (Chen & London, 1981; Moreno-Navarrete et al., 2017). However, in spite of a short-term treatment of adipocytes, effects elicited by hemin on lipolysis were relatively high. It can be supposed that hemin action was not associated with deterioration of cell viability. This assumption is supported by results showing that formazan formation from MTT, which reflects cell viability, did not significantly differ between the control cells and treated with hemin. Moreover, the action of insulin, DB-cAMP, forskolin and DPCPX was unchanged in the presence of hemin, which further confirms that cell functionality is preserved.

The adipocytes are known to store triglycerides, which undergo decomposition to glycerol and NEFA. These compounds, after release from the adipocytes, are the relevant source of energy for other kinds of cells (Frühbeck et al., 2014). However, an excessive release of glycerol and NEFA leads to pathological conditions in the whole organism. *In vivo* studies demonstrated that epinephrine-induced exaggerated release of glycerol largely contributes to insulin resistance and hyperglycemia. These pathological changes were reversed as a result of genetically induced aquaporin 7 deficiency (the main channel through which glycerol is released from the adipocytes) and the resulting diminished glycerol release (Maeda, Funahashi & Shimomura, 2008; Raje et al., 2020). Elevated blood levels of NEFA are also strongly associated with insulin resistance and type 2 diabetes. On the other hand, lowering of NEFA levels exerts an opposite effect and improves insulin action (Boden, 2011; Oh et al., 2018). In the present study, hemin was shown to markedly decrease lipolysis stimulated by epinephrine, which is associated with reduced release of glycerol and NEFA from adipocytes. This suggests that hemin may ameliorate consequences of the excessive release of glycerol and NEFA.

The present study was shown that hemin directly affects adipocyte metabolism. However, apart from lipid storage, fat cells play also a relevant endocrine role secreting various adipokines. These adipokines have multiple regulatory functions in the whole organism. Therefore, it would be interesting to explore the effects of hemin on adipokine secretion in the future.

## Conclusions

Results of the present study have shown that short-term exposure of the primary rat adipocytes to hemin decreases the lipolytic response of these cells to adrenergic stimulation. Similar effects were found for physiological (epinephrine) and pharmacological (isoproterenol) stimuli. Moreover, hemin was demonstrated to reduce lipolysis also in the presence of a high concentration of glucose. Given that supraphysiological concentrations of glucose are associated with an excessive lipolysis, our results indicate that this effect may be markedly alleviated by hemin. It was also revealed that hemin deepened the anti-lipolytic action of insulin, most likely due to changes in epinephrine action. The results concerning lipogenesis and lactate release indicate that these processes are unchanged after short-term exposure of the adipocytes to hemin. Our findings indicate that hemin action in the adipocytes is associated with reduced lipid release from these cells, also in the presence of supraphysiological concentration of glucose.

## Supplemental Information

10.7717/peerj.12092/supp-1 Supplemental Information 1 Raw data

## Additional Information and Declarations

### Data Availability

The following information was supplied regarding data availability: Raw data is available in the Supplementary File.
